# Climate change and infectious disease: A prologue on multidisciplinary cooperation and predictive analytics

**DOI:** 10.3389/fpubh.2023.1018293

**Published:** 2023-01-20

**Authors:** Kenneth B. Yeh, Falgunee K. Parekh, Illich Mombo, Joseph Leimer, Roger Hewson, Gene Olinger, Jeanne M. Fair, Yijun Sun, John Hay

**Affiliations:** ^1^MRIGlobal, Gaithersburg, MD, United States; ^2^EpiPointe LLC, Cary, NC, United States; ^3^CIRMF, Franceville, Gabon, Central African Republic; ^4^UK Health Security Agency, Salisbury, United Kingdom; ^5^London School of Hygiene and Tropical Medicine, London, United Kingdom; ^6^Los Alamos National Laboratory, Los Alamos, NM, United States; ^7^Jacobs School of Medicine and Biomedical Sciences, Buffalo, NY, United States

**Keywords:** climate, infectious disease, cooperative engagement, biosecurity, predictive analysis

## Abstract

Climate change impacts global ecosystems at the interface of infectious disease agents and hosts and vectors for animals, humans, and plants. The climate is changing, and the impacts are complex, with multifaceted effects. In addition to connecting climate change and infectious diseases, we aim to draw attention to the challenges of working across multiple disciplines. Doing this requires concentrated efforts in a variety of areas to advance the technological state of the art and at the same time implement ideas and explain to the everyday citizen what is happening. The world's experience with COVID-19 has revealed many gaps in our past approaches to anticipating emerging infectious diseases. Most approaches to predicting outbreaks and identifying emerging microbes of major consequence have been with those causing high morbidity and mortality in humans and animals. These lagging indicators offer limited ability to prevent disease spillover and amplifications in new hosts. Leading indicators and novel approaches are more valuable and now feasible, with multidisciplinary approaches also within our grasp to provide links to disease predictions through holistic monitoring of micro and macro ecological changes. In this commentary, we describe niches for climate change and infectious diseases as well as overarching themes for the important role of collaborative team science, predictive analytics, and biosecurity. With a multidisciplinary cooperative “all call,” we can enhance our ability to engage and resolve current and emerging problems.

## Background

In 2021, the United Nations (UN) held the 26th Climate Change Conference of the Parties (COP26). This event pressed nations to urgently address the goals and objectives of the Paris Accord and the UN Framework Convention on Climate Change, within the stated UN Sustainable Development Goals. One common concern is that evolving weather patterns and increased globalization of trade and travel will expedite climate change impacts. Climate change and its effects on infectious diseases are a comprehensive threat and therefore any mitigation effort must be equally comprehensive. Multidisciplinary engagements need to increase, harnessing and reinforcing technology and creating new approaches and platforms to identify and track climate change impacts, as a step toward mitigating their effects.

The “Frontiers” research topic, to which this manuscript is a Prolog, aims to highlight the connection between climate change and infectious disease, focusing on scientists working at the local, regional and global level, where innovations in predictive analytics using artificial intelligence (AI) and machine learning (ML) tools can assess relevant variables of impact. AI and ML involve processes and algorithms that are able to simulate human intelligence, including perception, learning and problem solving. Despite these names, AI and ML are simply approaches to data processing that try to maximize modern computing resources and are often referred together as AI/ML. AI/ML outputs are algorithmic models that are uniquely able to handle complex problems with large amounts data inputs and outputs. AI/ML models are generally developed based on data collection, parametrization, and model learning/validation.

**Figure 1:**
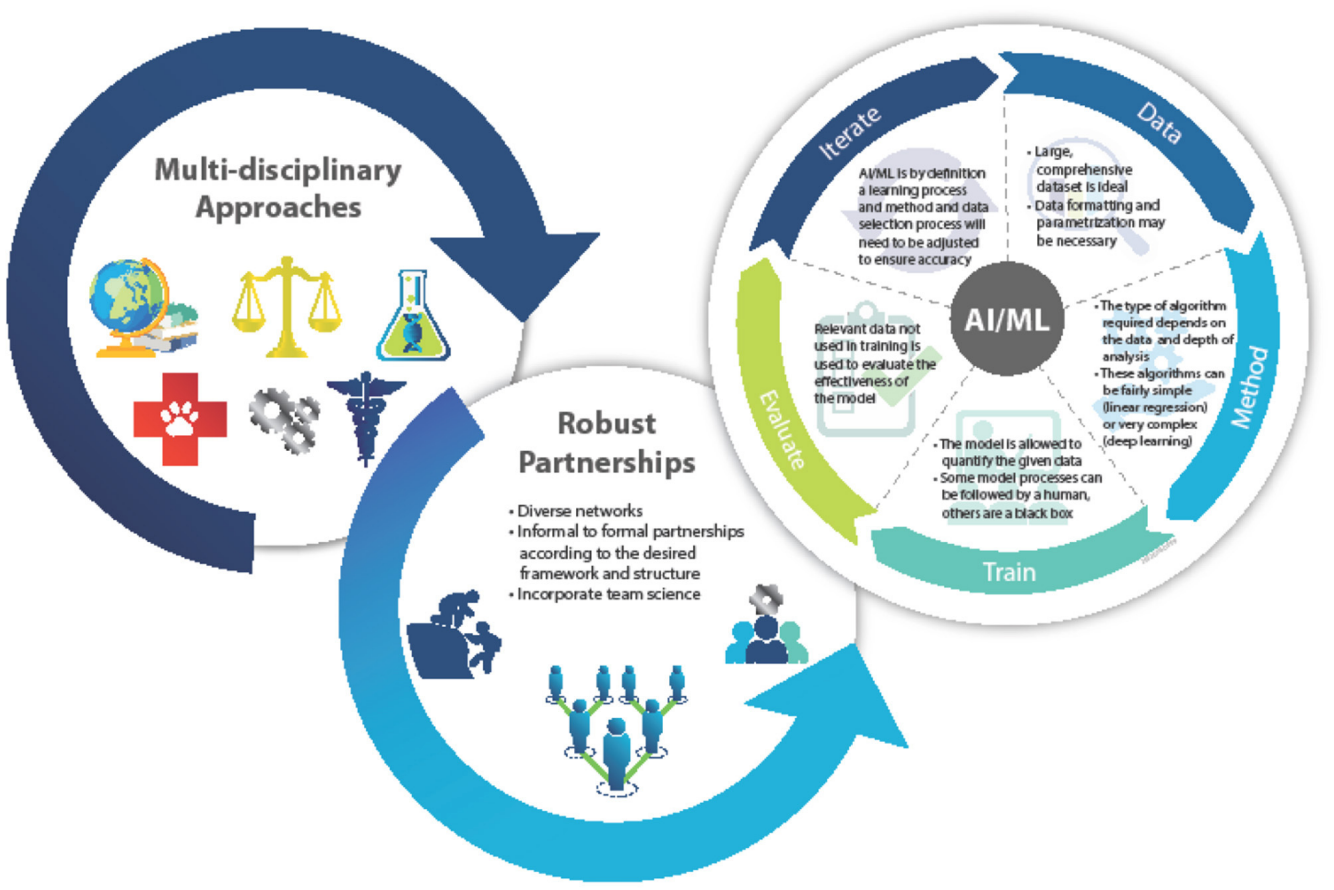
Depiction of the AI/ML modeling process. Defining the analysis and function ensures an accurate and precise product.

Climate change clearly impacts environmental biosecurity, requiring aspects of infectious disease surveillance, animal-human-plant health, ecology, and the environment to be studied in concert. In addition, robust networks, based on the above disciplines, offer agility, creativity, and trust which can accelerate engagements through familiarity and rapport among colleagues and peers. The elements of further multi-disciplinary and multi-sectoral collaborations are key to furthering outputs that can be effectively operationalized (Figure 1).

However, the variables that offer predictive insight into these phenomena are often difficult to track and manage, let alone analyze in real-time. The areas of interest, which are broad across and within disciplines that use AI/ML to link climate change and infectious disease, include studying human behaviors, exposure tracking, monitoring of personal protective equipment (PPE), rapid drug development, and monitoring of disease vectors induced by climate change. Recent reviews have discussed applying AI to infectious diseases and ML applications in microbiology as related to ecology, microbiome, infectious disease epidemiology, and drug discovery (1–3). Advances in AI/ML seek to make such predictive analyses more accessible and insightful.

## Infectious agents impacted by and impacting on environmental biosecurity

Assuming a One Health view, infectious diseases affect the ecology of hosts, vectors, and pathogens. *Vibrio cholerae* and *Yersinia pestis* are well studied examples of predictive modeling of water-borne and zoonotic diseases, respectively (4, 5). *Plant diseases* caused by pests and microbes impact environmental biosecurity, especially agriculture, and consequently food security across the world. Recent changes in weather toward warm and humid conditions have favored wheat blast outbreaks (6, 7), for example. Schistosomiasis, which is a classic neglected tropical disease, involves multiple organisms in a habitat where the intermediary snail population is impacted in drier climates and temperature changes (8). Clearly, infectious diseases that are spread to humans through non-human vectors are candidates for changes in geographic location based on changes in climate. These changes, seen currently as based on global rises in temperature, will potentially affect the ability of vectors to survive, both increasing and decreasing areas of disease prevalence. Below, we briefly discuss a sample of infectious diseases, including vector-borne diseases, that are intricately tied to the climate, the environment, animal reservoirs, and vectors.

1. **Sin Nombre virus** is a hantavirus, spread directly from the deer mouse (*P. maniculatis)* to humans in contact with these mice or their excreta, that causes hantavirus pulmonary syndrome (HPS); this is a rare but very serious disease, with a mortality rate of around 40%. In 1993, there was an HPS outbreak in the “Four Corners” region of the southwestern US. At the time, this was an “orphan” disease, but the “Hantavirus Study Group” quickly identified the virus and its vector. The relevance of this episode to this paper is that the reason for the outbreak was found to be a ten-fold increase in the vector population triggered by an El Nino winter (heavy Winter snow and Spring run-off) leading to abundant vegetation and food for *Peromyscus* (9). This outbreak in 1993, which is a classic example of the link between climate and infectious disease, was also the first use of newly-developed PCR technology, applied to identifying an unknown infectious disease. Researchers continued to study the dynamics of the virus and environment in the Four Corners region for several decades (10). Public health officials now regularly use climate data to predict rodent populations and the resulting risks for hantavirus outbreaks.
2. **Lyme Disease**, spread by *Ixodes scapularis* in the US, is the foremost tick-borne disease in North America and is caused by *Borrelia spp*. A lesser-known disease, also spread by *I. scapularis*, is ***Powassan virus disease*** (POW), which was first isolated in Powassan ON, Canada, from a child who died from encephalitis. While Lyme is hardly trivial, POW is a very serious and often fatal neurological disease, with long-term neurological sequelae in survivors (11). Ixodes ticks spend most of the time out in the environment, and so are sensitive to its moisture content (they must not get desiccated). In addition, *I. scapularis* feeds on small rodents, which are also dependent on food availability and therefore the climate. Climate modeling studies in the US predict future movement of *I. scapularis* westward and northward (12), leading to potential POW incidence increase in novel risk areas.
3. **Schistosomiasis** In 2008, Utzinger and his colleagues (13) published a discussion of the potential impact of climate change on schistosomiasis transmission in China. *Schistosoma japonicum* is one of the three major schistosome species and causes schistosomiasis in China. The parasite is spread to humans *via* contact with water in which an intermediate host snail, *Oncomelania hupensis*, has released sporocysts that develop into cercariae, the infectious form for humans. Their model, based on forecasted temperature ranges in which the schistosome was able to develop within the snail, predicted an expansion of schistosome transmission into currently non-endemic areas as a result of increased temperatures. Recently, an update was published (14) that confirmed the earlier predictions for China, showing areas in which transmission would increase. At the same time, those same likely climate changes should decrease transmission in other areas in which disease is currently prevalent. Similar predictions were made by De Leo and colleagues for Africa, identifying regions in which *S. haematobium* and *S. mansoni* transmission were likely to increase or decrease with climate change.
4. **Malaria** is the result of infection by *Plasmodium* species and is transmitted by female Anopheles mosquitoes. Six species are known to cause the disease in humans (15), among which *P. vivax* (SE Asia and western Pacific) and *P. falciparum* (Africa, SE Asia and western Pacific) are the most dangerous. In 2020, the WHO estimated ~241 million cases globally, with 629 000 deaths; the majority of cases and deaths are in sub-Saharan Africa. Elimination of malaria worldwide is an ultimate objective for the WHO. The Plasmodium life cycle has an exogenous sexual phase (sporogony) which occurs in several species of Anopheles mosquitoes, and an endogenous asexual phase (schizogony) which takes place in the vertebrate host (16). Climate changes have diverse effects on the Plasmodium life cycle and other vectors such as Culex which causes West Nile Fever, where extreme temperatures, humidity, wind, and precipitation affect vector distribution and abundance (17). For example, increased precipitation will expand the mosquito distribution area and extend the length of transmission season (18). For example, the number of human malaria infections in Eastern Africa, Nepal and Colombia (19–21) is rising, owing to warming of these regions, with an increase in competent malaria vectors at higher altitudes favoring disease transmission. Consequently, highland human populations that usually lack protective immunity are more vulnerable to severe malaria morbidity and mortality (22). Of course, although malaria transmission is climate-sensitive, like many other infections, it is a complex disease and changes in transmission cannot be attributed to climate alone (23, 24).
5. **Valley fever:** Valley fever is the common name for Coccidiodomycosis, a flu-like illness caused by a pathogenic fungus (*Cocccidiodes* spp). It is endemic in the southwestern United States and outbreaks often follow weather periods in which wet spells are followed by dry periods (10). The fungus grows in the wet period and spores that form when the land dries are easily spread through inhalation in dust clouds. A recent modeling analysis (25) predicts, in a high-warming scenario, that the disease will become endemic in an additional 5 states in the Western North US, with cases jumping by 50% by 2,100. Whether all of this will come to pass is uncertain, but the disease has recently spread to south central Washington State (26) and, with warming continuing through this century, at least some of this scenario seems likely to occur. In another nod to climate effects, a tsunami has also been suggested as a cause for the spread of this disease.

## Monitoring of disease vectors and climate resiliency

Collectively, vector-borne disease cases, for various reasons, have increased by 300% in the Americas over the last decade (27). Despite the already large impact of vector-borne diseases on global public health, morbidity and mortality will likely increase in the coming decades due to a now more rapidly-changing climate. Broad changes in temperature, wetness, and vegetation will all affect the prevalence of mosquitoes and the diseases they carry and drive them into entirely new geographic areas. Weather phenomena induced or exacerbated by climate change often coincide with increases in activity of disease vectors. For example, floods are some of the most devastating disasters induced by climate change in terms of physical damage and their ability to activate key disease vectors, such as mosquitos and wastewater exposure. Effectively predicting and monitoring these events is crucial in order to maintain global health. These phenomena are incredibly complex with many factors to consider but AI/ML can offer a pathway to effectively predicting floods and monitoring their effects (28). One example of AI/ML flood analytics in practice is from the company Ambiental Risk Analytics (https://www.ambientalrisk.com/).

## The importance of genome research

A panel discussion was recently published that emphasizes the value of genomic sequencing as a tool for addressing climate change and infectious disease with a One Health approach. One particular focus was how organisms shift and evolve during environmental change and its related complexities (29). When genomic sequencing tools are combined with risk and monitoring management efforts, an effective framework can be designed to illustrate priorities at multiple levels of impact (30). This combination of multi-disciplinary and multi-sectoral resources, as well as advances in predictive analytics using artificial intelligence and machine learning (AI/ML) tools, offers unique opportunities for effective approaches to exploring these problems.

Environmental disasters in which man-made infrastructures may impact climate factors also influence infectious disease outbreaks from resulting disruption of ecosystems, flooding, contamination, and displacement of human populations (31). These factors may cause increases (or decreases) in disease transmission as well as outbreaks which may produce chronic effects over time. Next generation studies, especially those that identify immediate risk factors and mitigation strategies combined with rapid genome identification will be increasingly valuable in the future.

## Partnerships: Need for multi-disciplinary engagement and building robust networks

Scientific collaboration networks can exist formally or informally and can focus on any given specific disease topic, a technology such as genomics and sequencing, or an emerging field such as ecoimmunology. One of the best ways of creating sustainable capabilities within countries is to connect researchers and the technical professionals together enabling cooperation or sharing expertise and information (32, 33). Research designed to tackle more global and complex challenges requires multidisciplinary teams that cross potentially more than a dozen fields in one project and bring together multiple technologies and data streams. This complexity in science teams requires a new look at how to better cooperate, collaborate, coordinate, and communicate (34). Informal networks are the connections, professional relationships, and source of contacts that scientists often leverage throughout their careers. These contacts include fellow researchers, peer colleagues, and mentor/mentees that connect at scientific conferences and related collaborations. These scientists maintain informal networks independently.

As science becomes more multidisciplinary across disparate fields, the breadth of the informal networks between researchers is becoming larger and more diverse. For example, infectious disease research requires understanding the ecology of an emerging or endemic infectious disease system where epidemiologists may work with meteorologists, sociologists, wildlife biologists, and geographers. With unlimited access between researchers through the internet, general connections are not endangered, however, trusted relationships and sustained connections are rarer. Cooperative engagement research is designed to build trusting and long-term relationships. The COVID-19 pandemic has led to an unprecedented amount of work and it was through the trusted collaborations that existed prior to the pandemic, that the critical initial data and information on the coronavirus was shared. Also, with the COVID-19 pandemic, most networks have had to move to become virtual networks. Having a low-cost virtual platform for connecting can help networks become sustainable into the future if and when funding ends. Despite these changes in connectivity and potential lowering costs to collaborate, resources specifically funding, remain a critical hurdle to sustain meaningful research.

During infectious disease outbreaks, both formal and informal networks are critical for a rapid and coordinated response. As it is often repeated, “if you exchange business cards on the first day of an outbreak, the pathogen has already won”. The return of investment for cooperative engagement programs became evident immediately in the 2020 COVID-19 pandemic (33). As the world moves into the future challenges of both a rapidly changing climate and increase threat of infectious diseases, scientific networks must be designed to be sustainable from the beginning and have a core vision of remaining sustainable.

## Predictive analytics—Artificial intelligence and machine learning

Each AI/ML model requires large and varied datasets for development, implementation and validation. For climate change and infectious disease surveillance, these data can be gathered from new research or existing datasets. Data are parametrized for use in the AI/ML so the relationships and key variables that model will investigate can be defined. Multiple parameters can be used at once, and often models need to be optimized simultaneously in a process referred to as multi-parameter optimization (35). The training for and validation of ML is generally performed using methods for supervised, unsupervised, or reinforcement machine learning. Supervised machine learning employs a fully labeled dataset that the model then uses to determine the relationship between inputs and outputs, while unsupervised machine learning uses unlabelled data to determine patterns and relationships among the dataset. Reinforcement machine learning uses a computer-video game method of trial and error to determine the best decision-making process. These processes also involve algorithm models to process data, where some common ML algorithms are naïve bayes, support vector machines, random forest, or artificial neural networks (36–38). To validate the AI/ML model its results are compared to a section of the original training dataset that was not inputted during the training process. Using these approaches AI/ML has shown itself to be invaluable at the intersection of climate change and infectious disease applications in surveillance, prediction, forecasting, research and development.

## Tracking human behaviours

One of the most prescient effects imposed by climate change is the temporary or permanent displacement of people due to climate induced disasters. As recent history has demonstrated, places that serve as nexus points for large traveling groups of people, such as ports of entry, refugee camps, and disaster locations are extremely potent vectors of new disease spread (39, 40). The term biosurveillance has broad meanings and different connotations depending on whether the context is animal, human, plant or microbes. Accounting for human behavior in an immediate fashion has been difficult due to the multitude of other factors involved; however, with advances in AI/ML analysis, this type of bio-surveillance can sometimes be performed rapidly and non-intrusively. Acceptable examples of such technology range from consumer marketing and internet search preferences to digital exposure tracking and PPE monitoring. At the same time, constant direct monitoring through national government also has its value but often varies substantially in quality, depending on political will and resource availability.

## Climate and disease forecasting

Vector-borne diseases are particularly susceptible to climate impacts, as they depend on dynamic ecosystems. Complex interactions between weather, hydrology, vegetation, and the surrounding ecosystem, as well as human-created habitats impacted by economics and infrastructure can cause vector activity to explode or collapse. Vector ecosystems also vary by geographic locations with predictability in one region having no predictive value in another region. There are many challenges in being able to forecast and predict short- and long-term changes of climate-driven infectious diseases. Integrating the diverse causes of changes in vector ranges and resulting transmission of infectious diseases into a more modular computational model that covers climate, environmental and vector changes is a complex task (41). The challenge is to have a fine-scale prediction of mosquito-borne diseases, for example, that is flexible enough to integrate disparate, imperfect data and generate new predictions on demand as climate models improve and adapt to new data. Being able to forecast and predict mosquito borne diseases, such as Dengue and Chikungunya virus will help align bio-surveillance efforts to track the progression of the disease into new territory. Combining climate and environmental science and disease epidemiology and ecology will be critical for knowing where infections might move in the future and how we can be better prepared to mitigate and track these advancing threats.

## Exposure tracking

Digital exposure tracking is a technology that came into wide use during the COVID-19 pandemic as a way to warn of potential exposure, provide contact tracing, and give out quarantine guidance. AI/ML was necessary in this instance to manage the need for rapid response times and large volumes of data that include tracking individual health information, contact between personal devices (i.e., cell phones), GPS, and cell towers. Digital exposure tracking also has higher accuracy than traditional exposure tracking that is largely based on human recollection. Using all of these data filtered through an AI/ML algorithm, authorities are able to effectively monitor the risk of infection and act quickly in order to mitigate any exposures (42, 43).

One example of successful implementation of digital exposure tracking during the COVID-19 pandemic is the approach implemented by Taiwan. At the beginning of the pandemic, the Taiwanese government rapidly created a system that allowed for rapid communication between government agencies and individuals allowing for the quantification of potential infection risk. With this system, the cases were able to be contained far below those predicted by initial fears (44). As infectious disease outbreaks shift between diseases, with the 2022 Monkeypox global outbreak being an example, the tracking technologies instituted for COVID-19, can be readily adapted for use in these new situations.

## PPE monitoring

With the demonstrated effectiveness of PPE in preventing the spread of disease, it is crucial to ensure that in high-risk areas the proper preventative measures are taken (45). AI/ML can be used to track PPE usage and/or monitor for potential exposure due to PPE failure through the use of machine vision. Machine vision allows for an AI/ML monitoring system to recognize and record visual stimuli, allowing for real time monitoring of the relevant parameters. This technology already has established uses in the area of hazardous workplace PPE monitoring. One example is AIM2 by agile labs (https://www.aim2.info/ppemonitoring/). This program is trained to recognize hard hats, safety vests, and goggles and give warnings if it detects such items are not in use. It also catalogs potential breaches for liability purposes.

## Rapid drug development

Climate change will affect the behavior and activity of disease reservoirs and vectors such as mosquitoes and ticks (46). This in turn will increase the likelihood for outbreaks of disease and/or the appearance of novel disease strains. The response to these potential disease events needs to involve a more rapid and comprehensive response than traditional drug development can provide. AI/ML (often referred to in a drug development context as *in silico* drug development) can provide the tools to overcome this challenge. AI/ML drug development can analyze existing drugs for effectiveness against new disease agent strains (47, 48), optimize new drugs for desired characteristics such as adsorption, distribution, metabolism, elimination, and toxicity (ADMET) (37), and develop completely novel treatments such as antibodies and vaccines (49, 50).

A specific example of how AI/ML speeds up the drug development process is demonstrated by the AI/ML biotech firm AbCellera partnering with Eli Lily to rapidly develop anti-COVID antibody treatments (51). AbCellera used ML to identify therapeutic antibodies from a convalescent SARS-CoV-2 patient by screening 5.8 million peripheral blood cells (PBMCs) to produce 440 high confidence antibody sequences. ML was further used to select the promising antibodies that appeared in high frequency across the dataset and eventually narrowed down the field to 24 lead candidates that were then tested for *in vitro* study. The ML process took place in under a month and resulted in the production of LY-CoV555 which showed success in non-human primates (NHPs) at neutralizing the virus.

## Conclusions

Even under the most optimistic projections, the climate will be noticeably variable over a period of decades or years rather than centuries, pushing us into a constantly changing public health landscape in part due to increased data collection. We are faced with a new “non-equilibrium” global health crisis. Climate change clearly impacts infectious disease and biosecurity generally, requiring aspects of bio-surveillance, animal-human-plant health, ecology, and environment to be studied in concert. With limited resources and political will, the reactive response posture nevertheless remains a mainstay of the national and international approach to infectious diseases for humans, animals, and plants. Otherwise, the continual reactive response to infectious disease research severely limits our ability to prepare for, detect, and respond to any outbreaks. A successful top-down approach requires unprecedented national multi-agency to multi-sectoral and multi-disciplinary cooperation. Climate change does not choose ownership, so this challenge requires world-wide contribution and engagement. On one hand, COVID-19 demonstrated inter-governmental and multi-national cooperation to an urgent and compelling extent but, at the same time, climate change is so complex that future challenges may neither be evident nor approachable with current tools.

Moving from disparate data sets—to information—to actionable decisions is now possible with predictive analytics methods. Converging data, analytic approaches, and technology offers ways to identify threats, close gaps, and remediate threats. A proactive data driven analytical method that predicts biothreats before they become a problem is now both feasible and necessary. Work demonstrating successful outputs and returns on investments in predictive analytics, such as design-build-learn-train models, as well as public-private partnerships and how such work may be pivoted to mitigate climate change impacts, will be especially relevant.

Managing public health emergencies effectively requires several practical issues to be resolved, including common standards for data collection, common data handling systems and interoperability, and common public health IT infrastructure. This applies within a particular country but, given the international nature of climate effects on public health, it also needs to apply globally among countries. Many high income countries have developed common national public health standards but these do not always mesh with those from other countries (52) and many countries still have fragmented public health systems. The World Health Organization (WHO) is the most visible entity involved in global health data collection and its “SCORE” system is one useful approach to assisting member states strengthen health data collection and sharing. Nevertheless, not every country seems prepared to operate through the WHO, which lessens its usefulness and potential impact.

Given the lack of global international agreement on next steps to counter climate change, it will be difficult to gain momentum on what to do about its effects.

## Data availability statement

The original contributions presented in the study are included in the article/supplementary material, further inquiries can be directed to the corresponding author.

## Author contributions

KY, FP, JF, RH, YS, and JH developed this concept as related to their research topic call. JL, IM, and GO also contributed content to this manuscript. All authors reviewed and agreed on the final submission.
